# The Role of Anticoagulants and Antiplatelets in Reducing Mortality in COVID-19 Patients: A Systematic Review and Meta-Analysis of Studies Reporting Adjusted Data

**DOI:** 10.7759/cureus.45749

**Published:** 2023-09-22

**Authors:** Muskaan Doulat Ram, Muhammed Umer, Ishani Jayantibhai Trada, Salman J Khan, Laiba Imran, Tayyaba Rehan, Warda Hassan, Faiqa Zafar, Sufyan Razak, Tooba Laeeq, Parisa Aijaz, Zainab Majid

**Affiliations:** 1 Internal Medicine, Dow University of Health Sciences, Karachi, PAK; 2 Internal Medicine, American University of Barbados, Bridgetown, BRB; 3 Internal Medicine, Mayo Clinic, Jacksonville, USA; 4 General Surgery, New York Institute of Osteopathic Medicine, New York, USA; 5 Oncology, Johns Hopkins University School of Medicine, Baltimore, USA; 6 Internal Medicine, Kirk Kerkorian School of Medicine at University of Nevada, Las Vegas (UNLV), Las Vegas, USA; 7 Internal Medicine, Charleston Area Medical Center, Charleston, USA

**Keywords:** mortality, antiplatelets, anticoagulants, severe acute respiratory syndrome, covid-19

## Abstract

The coronavirus disease 2019 (COVID-19) is associated with prolonged prothrombin time (PT), active partial thromboplastin time (aPTT), and increased D-dimer levels. Therefore, we aim to investigate if anticoagulants (AC) and antiplatelet (AP) therapy play a role in mitigating COVID-19 and its associated thrombosis along with its effect on the mortality rate, the need for mechanical ventilation, and the risk of hospital admission. Electronic databases were searched from their inception to July 19, 2022. The studies were divided into two groups: Group A (any dose of AC/AP versus no AC/AP) and Group B (therapeutic dose of AC (tAC)/AP versus prophylactic dose of AC (pAC)/AP). Review Manager (RevMan) version 5.4.1 (The Nordic Cochrane Centre, The Cochrane Collaboration, Copenhagen, Denmark) was used for all statistical analyses. Adjusted data ratios were extracted from all included studies and pooled using the random effects model. A total of 33 studies were taken for the analysis of two groups (Group A: 285,065 COVID-19-positive patients, Group B: 2,421 COVID-19-positive patients). Overall analysis in Group A showed that the AC/AP group had a low risk of mortality in COVID-19 patients compared to the control group (risk ratio (RR): 0.77, 95% confidence interval (CI): 0.69-0.86). There was no significant difference in the need for mechanical ventilation (RR: 0.80, 95% CI: 0.60-1.08) and hospital admission (RR: 1.12, 95% CI: 0.78-1.59) between the AC/AP and no AC/AP group. Alongside, in Group B, tAC/AP did not demonstrate a significant decrease in mortality as compared to pAC/AP (RR: 0.62, 95% CI: 0.37-1.06). Treatment with AC and AP drugs can significantly decrease the mortality rate in COVID-19-infected patients, while AC also significantly reduces the need for mechanical ventilation.

## Introduction and background

The coronavirus disease 2019 (COVID-19) has a very high mortality rate, and over the course of two and a half years, the virus has claimed over 6,000,000 lives globally [1]. While the disease is mainly known for its associated pulmonary complications, it has also been linked with dangerous cardiovascular complications, mainly thromboembolic events [2]. The hypercoagulable state caused by the virus is corroborated by prolonged prothrombin time (PT) and active partial thromboplastin time (aPTT), along with increased D-dimer levels [3]. The coagulopathy seen in COVID-19 patients manifests in the form of venous and arterial thromboembolism and is associated with a poor prognosis [3].

Considering the thrombogenic effects of COVID-19, it is plausible that anticoagulation benefits patients by reducing the risk of thromboembolism while also ultimately decreasing the risk of mortality [4]. Therapeutic anticoagulation (tAC) and prophylactic anticoagulation (pAC) have both been linked with reduced mortality rates and a decreased need for intubation in COVID-19 patients [5]. Since platelets play an important role in clotting and contribute to thrombus formation, the use of antiplatelet agents such as aspirin and dipyridamole in the management of COVID-19 has also proven to be beneficial [6].

Previous meta-analyses have been carried out on unadjusted data [7]. COVID-19 infection is associated with unique laboratory findings such as thrombocytopenia with elevated fibrinogen and fibrin D-dimer; all these laboratory abnormalities are associated with poor outcomes. A fine balance of thrombotic prophylaxis is required to reduce mortality and morbidity without increasing the risk of bleeding [7]. Studies have shown that adjusted data is more robust than non-adjusted data as it takes into account and minimizes the effect of cofounders [8]. Our meta-analysis comprises adjusted data from studies evaluating the efficacy of anticoagulants (AC) and antiplatelet (AP) agents in the management of COVID-19. We aim to investigate if AC and AP therapy play a role in mitigating COVID-19 and the thrombosis associated with it while also evaluating its effect on the COVID-19 mortality rate, the need for mechanical ventilation, and the risk of hospital admission.

## Review

Methods

This study is conducted as per the Preferred Reporting Items for Systematic Reviews and Meta-Analyses (PRISMA) guidelines [9].

Literature Search

Databases such as PubMed, Medline, Google Scholar, Medrix, and Cochrane CENTRAL were used from their inception to August 19, 2022, with the help of a search string using the following keywords: COVID-19 OR Coronavirus OR SARS-CoV-2 OR severe acute respiratory syndrome coronavirus 2 AND Anticoagulants OR Antiplatelets OR Aspirin OR Warfarin OR Coumadin OR Heparin OR Clopidogrel OR Dabigatran OR Ticagrelor OR Prasugrel OR Acetylsalicylic acid OR Rivaroxaban OR Heparin OR LMWH OR Cilostazol OR Apixaban. Furthermore, we looked for any relevant studies or previous meta-analyses for any grey literature. A detailed literature search is shown in the Appendices.

Inclusion Criteria

The criteria for the included study were as follows: (a) participants aged ≥18 years who were screened and tested positive for COVID-19, (b) any dose of AC/AP compared with a placebo, (c) a therapeutic dose of AC/AP compared with a prophylactic dose, (d) reported at least mortality outcome, and (e) results reported after adjusting for covariates. We accepted the primary investigators’ definitions of therapeutic and prophylactic doses.

Studies not meeting the above criteria, review articles, case reports, animal studies, editorials, and perspective communications were not included. Studies that did not report outcomes for adjusted data and were in languages other than English were also excluded.

Study Selection and Quality Assessment

Two reviewers independently performed an online search. All the articles were retrieved into the EndNote Reference Library software X4 to check for repeats. The reviewers assessed and selected the articles that matched the inclusion criteria. At first, articles were shortlisted based on title and abstract, and then, the full text of the article was reviewed to confirm the study’s relevance. Another reviewer was consulted for any discrepancies.

The modified Cochrane Collaboration’s risk of bias tool and the Newcastle-Ottawa Scale were used for assessing the quality of randomized control trials and observational studies, respectively [10,11]. The Cochrane tool focuses on different domains ranging from design to conduct, to reporting of the control trials, while the Newcastle-Ottawa Scale focuses on three areas: selection, comparability, and ascertainment.

Data Extraction, Groups, and Outcomes

Baseline demographics such as age, gender, and comorbidities (diabetes mellitus (DM), hypertension, chronic obstructive pulmonary disease (COPD), atrial fibrillation, heart failure, ischemic stroke, chronic kidney disease, coronary artery disease, and smoking history) and details of anticoagulation were extracted from each study. Details of variables that were adjusted in each study were also extracted.

The studies were divided into two groups: Group A, which consisted of studies where any dose of AC or AP (experimental) was compared to no AC or AP (control), and Group B, which consisted of studies that compared the therapeutic dose of AC or AP (tAC/AP) (experimental) to the prophylactic dose of AC or AP (pAC/AP) (control).

Only those outcomes were considered that were analyzed based on adjusted data. Adjusted data helps us in homogenizing the study population and overcomes the problem of missing or uneven data. Mortality, need for mechanical ventilation, and risk of hospital admission were reported for AC/AP versus no AC/AP (Group A), and only mortality outcome was reported for tAC/AP versus pAC/AP (Group B).

Statistical Analysis

Review Manager (RevMan) version 5.4.1 (The Nordic Cochrane Centre, The Cochrane Collaboration, Copenhagen, Denmark) was used for all statistical analyses. Adjusted data ratios were extracted from all included studies and pooled into RevMan using the random effects model. Risk ratio (RR) along with 95% confidence interval (CI) were calculated and reported for all outcomes. Subgroup analysis was done based on AC and AP type, and a p-value lower than 0.05 was considered significant.

The leave-one-out sensitivity analysis was carried out on the involved outcomes: mortality, ventilation, and hospital admission risk. In addition, heterogeneity was assessed with the help of Higgins I^2^; 25%-50% was considered to be mild heterogeneity, 50%-75% was considered moderate heterogeneity, and >75% was considered significant heterogeneity [12].

Results

Literature Search Results

**Figure 1:**
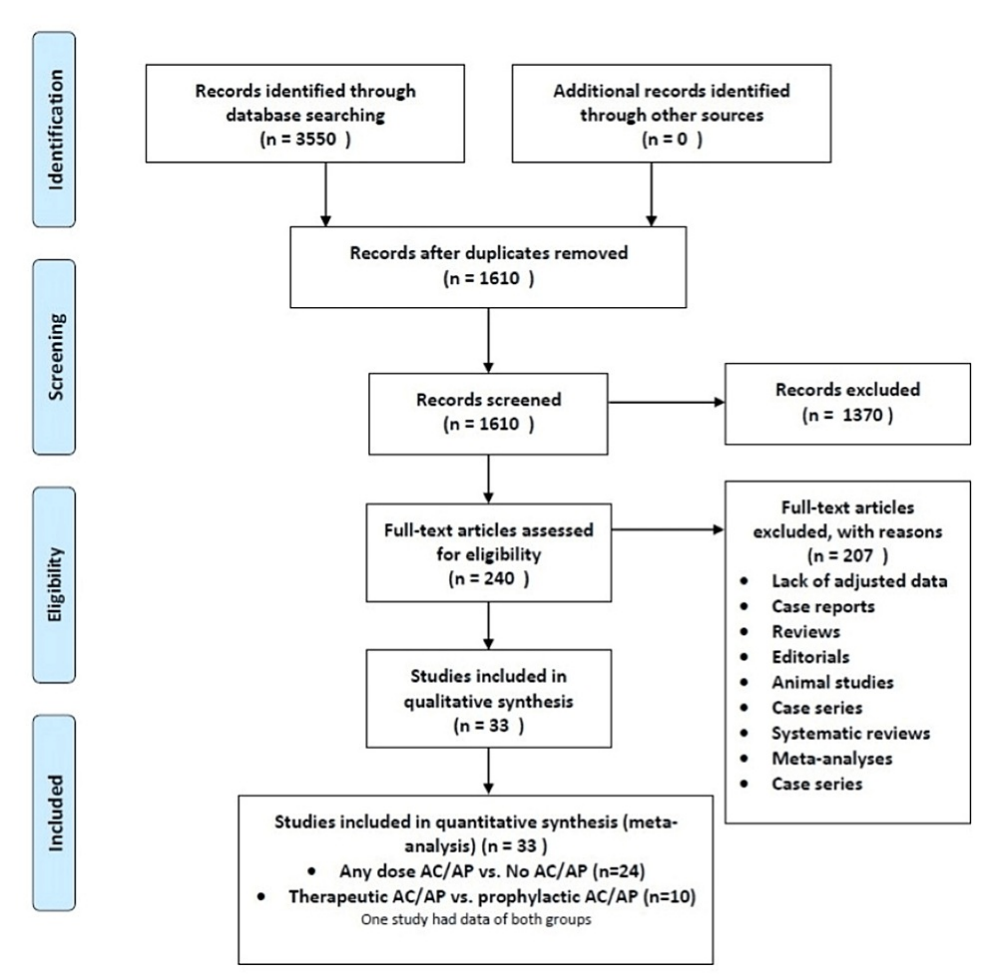
PRISMA flowchart showing the summary of the literature search PRISMA: Preferred Reporting Items for Systematic Reviews and Meta-Analyses, AC: anticoagulant, AP: antiplatelet

A total of 3,550 articles were recruited from all the databases, and 240 studies were shortlisted to satisfy the eligibility criteria. After screening the 240 studies for adjusted data and outcomes, 33 studies [13-45] were taken to analyze two groups. The detailed search is summarized in the PRISMA flowchart (Figure 1).

Study Characteristics

Group A (any dose of AC/AP versus no AC/AP) of the meta-analysis included 24 studies with a total of 285,065 COVID-19-positive patients: 190,224 in the AC/AP arm and 94,841 in the placebo (no AC/AP) arm. The baseline characteristics of the studies are mentioned in Table 1.

**Table 1:** Baseline characteristics for Group A (any dose AC/AP versus no AC/AP) studies Group A consisted of studies where any dose of AC or AP (experimental) was compared to no AC or AP (control). AC: anticoagulant, AP: antiplatelet, IQR: interquartile range, SD: standard deviation, DM: diabetes mellitus, CKD: chronic kidney disease, CAD: coronary artery, COPD: chronic obstructive pulmonary disease, pAC: prophylactic dose of AC, tAC: therapeutic dose of AC

Study name	Study location	Age (years) (median (IQR)/mean ± SD)	Male gender (%)	AC or AP group/control group (number)	Hypertension (number (%))	DM (number (%))	Atrial fibrillation (number (%))	Heart failure (number (%))	Ischemic stroke (number (%))	CKD (number (%))	CAD (number (%))	COPD (number (%))	Current smoker (number (%))
Paranjpe et al. (2020) [13]	New York	-	-	786/1,987	-	-	-	-	-	-	-	-	-
Russo et al. (2020) [14]	Italy	67.7 ± 15.2	115 (59.9)	81/111	111 (57.8)	42 (21.9)	24 (12.5)	20 (10.4)	16 (8.3)	12 (6.2)	26 (13.5)	26 (13.5)	-
Tremblay et al. (2020) [15]	New York	56.6 ± 18.2	2,067 (54.8)	913/2,859	-	-	-	-	-	-	-	-	155 (4.1)
Nadkarni et al. (2020) [16]	New York	65 (53-77)	2,457 (66)	2,859/1,530	1,526 (34.8)	991 (22.6)	298 (6.8)	362 (8.3)	-	493 (11.3)	541 (12.4)	216 (4.9)	-
Shankaranarayanan et al. (2020) [17]	-	67 (59-75)	57 (71)	350/152	-	-	-	-	-	-	-	-	-
Rivera-Caravaca et al. (2020) [18]	Ecuador, Germany, Italy, and Spain	AC group: 81 (75-87), control group: 83 (74-88)	129 (59.1)	109/109	179 (82.1)	68 (31.2)	10 (4.6)	-	42 (19.3)	33 (15.1)	31 (14.2)	40 (18.3)	9 (4.1)
Rentsch et al. (2020) [19]	United States	68.3 (58.2-75)	8,010 (93.4)	4,303/4,273	67.80	42.90	-	10.90	-	19.50	2.7	15.20	-
Denas et al. (2020) [20]	Italy	75-84 (44.9%)	2,546 (54.2)	651/4,046	87.70	23.60	-	16.60	14	8.4	-	-	-
Flam et al. (2020) [21]	Sweden	73.6 (7.6)	8,476 (60.3)	103,703/36,875	-	-	-	25.60	17	-	-	-	-
Meizlish et al. (2021) [22]	United States	>60 (58.7%)	50.1	1,624/1,956	-	-	-	-	-	-	-	-	-
Di Castelnuovo et al. (2021) [23]	Italy	68 (57-79)	1,555 (60.4)	1,804/770	52.30	21.40	-	-	-	-	-	-	-
Ionescu et al. (2020) [24]	United States	74 ± 15	69 (54)	pAC: 47, tAC: 67, no AC: 13	78	51	14	24	21	22	25	-	-
Falcone et al. (2020) [25]	Italy	70 (57-80)	76.2	244/838	41.20	18.40	-	-	-	5.70	-	7.80	-
Ionescu et al. (2021) [26]	Italy	64.5 ± 17.0	48.5	pAC: 2,121, tAC: 998, no AC: 361	-	-	-	-	-	-	-	-	-
Chow et al. (2021) [27]	United States	55 (41-66)	59.2	98/314	78.60	55.10	-	-	-	-	34.70	-	-
Al-Samkari et al. (2021) [28]	United States	61 (53-71)	64.5	384/2,425	-	-	-	-	-	-	-	-	-
Haji Aghajani et al. (2021) [29]	Iran	61.640 ± 17	54.89	AC: 336, no AC: 655	41.07	30.58	-	-	-	10.29	19.58	8.78	-
Rivera-Caravaca et al. (2021) [30]	United Kingdom	67.30 ± 15.43	13,416 (51.6)	13,003 /13,003	18,707 (71.9)	9,846 (37.9)	12,498 (48.1)	7,564 (29.1)	5,286 (20.3)	8,842 (34)	-	4,763 (18.3)	-
The OpenSAFELY Collaborative et al. (2021) [31]	United Kingdom	71 (66-75)	79.2	52,416/18,048	21,851 (41.7)	6,340 (12.09)	-	5,624 (10.7)	1,029 (2)	8,431 (16.1)	-	5,216 (10)	3,894 (7.4)
Vaughn et al. (2021) [32]	Michigan	64 (52-75)	52.2	1,127/162	157 (71.7)	-	-	-	-	68 (31.1)	-	-	-
Spiegelenberg et al. (2021) [33]	Netherlands	76 (72-82)	71	856/964	112 (59)	52 (27)	-	40 (21)	37(19)	-	52 (27)	-	-
Lund et al. (2022) [34]	Denmark and Sweden	Denmark: 72 (59-82), Sweden: 60 (47-73)	Denmark: 50, Sweden: 56	Denmark: 771/921, Sweden: 1,167/701	-	-	Denmark: 15 (2), Sweden: 12 (1)	Denmark: 57 (7), Sweden: 56 (5)	Denmark: 58 (8), Sweden: 28 (2)	Denmark: 11 (1), Sweden: 21 (2)	Denmark: 188 (24), Sweden: 165 (14)	-	Denmark: 55 (10), Sweden: -
Buenen et al. (2021) [35]	Netherlands	72	64	110/387	87 (79)	29 (26)	-	-	-	35 (32)	-	-	-
Hara et al. (2021) [36]	Japan	67 (56-76)	71.95	367/1,381	166 (45.2)	121 (34.8)	-	10 (2.7)	19 (5.2)	2 (0.5)	-	46 (12.5)	158 (43.1)

Group B of the meta-analysis includes 10 studies with a total of 2,421 COVID-19-positive patients: 929 in the therapeutic arm and 1,492 in the prophylactic arm. The baseline characteristics of the included studies are mentioned in Table 2.

**Table 2:** Baseline characteristics for Group B (therapeutic dose of AC/AP versus prophylactic dose of AC/AP) studies Group B consisted of studies that compared the therapeutic dose of AC or AP (tAC/AP) (experimental) to the prophylactic dose of AC or AP (pAC/AP) (control). AC: anticoagulant, AP: antiplatelet, IQR: interquartile range, SD: standard deviation, DM: diabetes mellitus, COPD: chronic obstructive pulmonary disease, CVD: cardiovascular disease, pAC: prophylactic dose of AC, tAC: therapeutic dose of AC

Study name	Study location	Age (years) (median (IQR)/mean ± SD)	Male gender (number (%))	Therapeutic/prophylactic group (number)	DM (number (%))	Pulmonary disease (number (%))	Kidney disease (number (%))	COPD (number (%))	Hypertension (number (%))	Heart failure (number (%))	Stroke (number (%))	CVD (number (%))
Ferguson et al. (2020) [37]	-	Therapeutic: 65 (56-73), prophylactic: 63 (52-71)	78 (55.3)	46/95	34 (24.1)	-	-	-	-	-	-	-
Pesavento et al. (2020) [38]	Padua, Italy	70 (57-81)	181 (55.9)	84/240	-	-	-	-	-	-	-	-
Motta et al. (2020) [39]	Fairfield	64.7 (18.1)	220 (58.8)	75/299	118 (31.6)	94 (25.1)	40 (10.7)	-	-	-	-	-
Bolzetta et al. (2020) [40]	Italy	pAC: 84.1 ± 11.1, tAC: 87.1 ± 8.0	pAC: 13 (54), tAC: 40 (70)	24/57	pAC: 22.8, tAC: 29.2	-	-	pAC: 17.5, tAC: 8.3	pAC: 66.7, tAC: 61.4	pAC: 3.5, tAC: 4.2	pAC: 7, tAC: 4.2	-
Meizlish et al. (2021) [22]	United States	-	-	191/191	-	-	-	-	-	-	-	237
Martinelli et al. (2021) [41]	-	59 (49-67)	181 (65.1)	127/151	-	-	-	-	-	-	-	-
Trinh et al. (2020) [42]	New York	59.6 ± 13.2	161 (66)	161/83	90 (36.9)	-	24 (9.8)	10 (4.1)	122 (50)	-	-	-
Jonmarker et al. (2020) [43]	Sweden	61 (52-69)	(82.2)	37/67	-	-	-	-	-	-	-	-
Matli et al. (2021) [44]	Lebanon	Prophylactic: 59.69 ± 17.04, therapeutic: 62.55 ± 15.8	11 (13.4)	31/51	Prophylactic: 10 (19.6), therapeutic: 8 (25.8)	-	4 (4.9)	Prophylactic: 0 (0), therapeutic: 1 (3.2)	Prophylactic: 20 (39.2), therapeutic: 14 (45.2)	Prophylactic: 2 (3.9), therapeutic: 1 (3.2)	-	Prophylactic: 1 (2), therapeutic: 1 (32)
Hoogenboom et al. (2022) [45]	United States	Prophylactic: 56 (48-67), therapeutic: 63 (53-72)	Prophylactic: 66.4, therapeutic: 72.8	153/158	Prophylactic: 49 (31), therapeutic: 43 (28)	-	Prophylactic: 11 (7), therapeutic: 14 (9)	Prophylactic: 4 (3), therapeutic: 16 (10)	Prophylactic: 66 (42), therapeutic: 83 (54)	Prophylactic: 10 (6), therapeutic:11 (7)	-	Prophylactic: 17 (11), therapeutic: 26 (17)

Quality Assessment and Publication Bias

**Figure 2:**
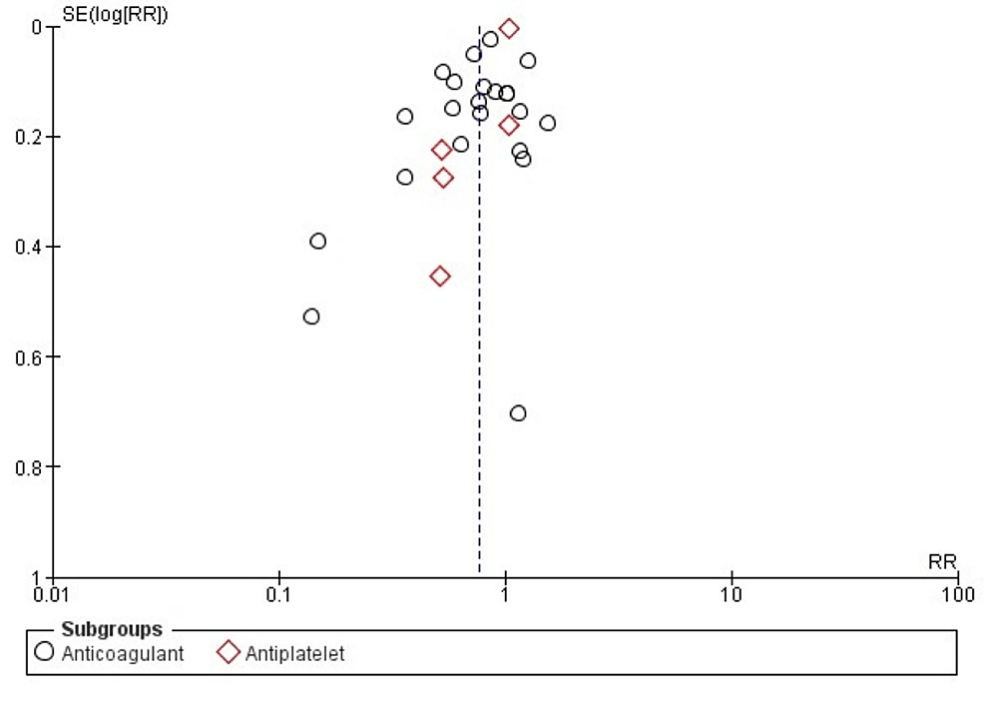
Funnel plot of publication bias

The quality assessment showed that the included studies in both groups had a low to moderate risk of bias. Visual assessment of the funnel plot showed no publication bias, which was also confirmed by Egger’s regression (p = 0.21) (Figure 2).

Results of Meta-Analysis for Group A (Any Dose of AC/AP Versus No AC/AP)

All-cause mortality: All 24 studies reported mortality outcomes. Overall analysis showed that the AC/AP group had a low risk of mortality in COVID-19 patients compared to the control group (RR: 0.77, 95% CI: 0.69-0.86; p < 0.00001; I^2^ = 93%).

**Figure 3:**
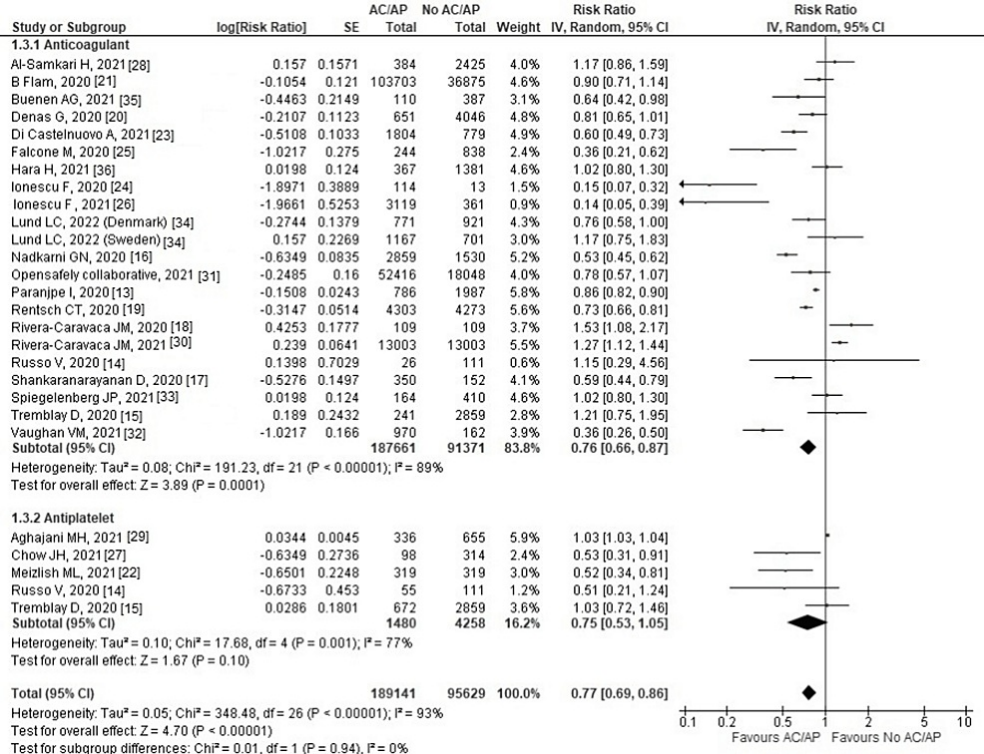
Forest plot of all-cause mortality outcome Red squares and their corresponding lines are the point estimates and 95% CI per each study. Black diamonds represent the pooled effect estimate. IV: inverse variance, SE: standard error, CI: confidence interval, AC: anticoagulant, AP: antiplatelet

On subgroup analysis, the AC subgroup showed a statistically significant decrease in the risk of mortality (RR: 0.76, 95% CI: 0.66-0.87; p < 0.0001; I^2^ = 89%), whereas in the AP subgroup, a non-significant decrease in mortality was seen compared to the control group (RR: 0.75, 95% CI: 0.53-1.05; p = 0.10; I^2^ = 77%) (Figure 3).

On sensitivity analysis, removing any study did not significantly change the overall results and heterogeneity.

Need for mechanical ventilation: Out of 24 studies, only four reported mechanical ventilation as one of their outcomes. There was no significant difference in the need for mechanical ventilation between the AC/AP group and the no AC/AP group (RR: 0.80, 95% CI: 0.60-1.08; p = 0.15; I^2^ = 62%).

**Figure 4:**
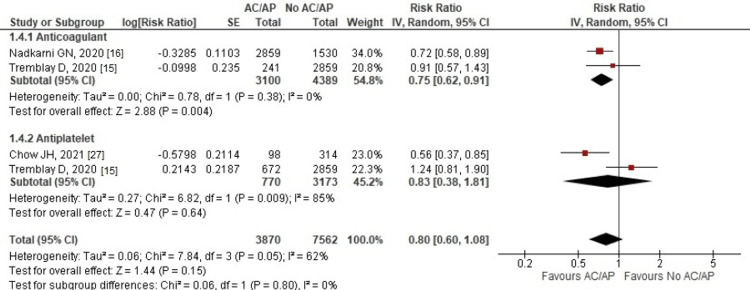
Forest plot of mechanical ventilation Red squares and their corresponding lines are the point estimates and 95% CI per each study. Black diamonds represent the pooled effect estimate. IV: inverse variance, SE: standard error, CI: confidence interval, AC: anticoagulant, AP: antiplatelet

On subgroup analysis, a significant decrease was noted in the AC subgroup (RR: 0.75, 95% CI: 0.62-0.91; p = 0.004; I^2^ = 0%), but the AP subgroup showed no significant decrease (RR: 0.83, 95% CI: 0.38-1.81; p = 0.64; I^2^ = 85%) (Figure 4).

On sensitivity analysis, removing the study by Tremblay et al. (2020) [15] significantly changed the overall heterogeneity from 62% to 15% and turned the results significant (RR: 0.71, 95% CI: 0.58-0.87; p = 0.001; I^2^ = 15%).

**Figure 5:**
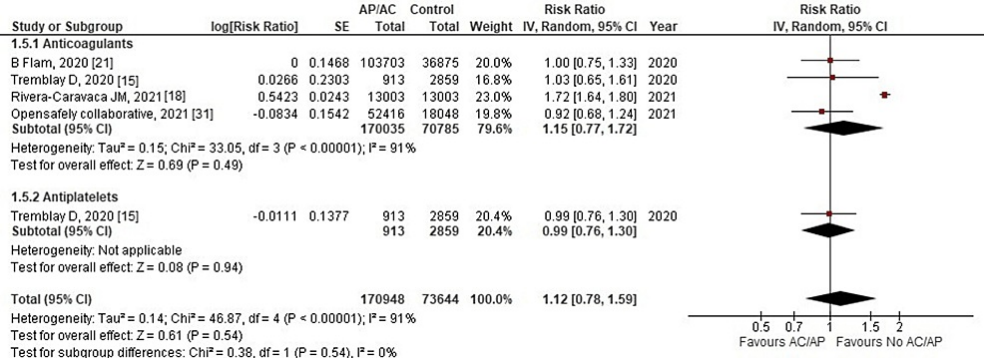
Forest plot of hospital admission Red squares and their corresponding lines are the point estimates and 95% CI per each study. Black diamonds represent the pooled effect estimate. IV: inverse variance, SE: standard error, CI: confidence interval, AC: anticoagulant, AP: antiplatelet

Risk of hospital admission: Only four studies reported hospital admission as one of their outcomes. The results did not show a significant difference between the AC/AP group and the control group (RR: 1.12, 95% CI: 0.78-1.59; p = 0.54; I^2^ = 91%) (Figure 5).

Removing the study by Rivera-Caravaca et al. (2021) [18] reduced the heterogeneity level from 91% to 0%, but the results remained insignificant (RR: 0.98, 95% CI: 0.84-1.14; p = 0.78; I^2^ = 0%).

Results of Meta-Analysis for Group B (Therapeutic Dose of AC/AP (tAC/AP) Compared With Prophylactic Dose of AC/AP (pAC/AP))

**Figure 6:**
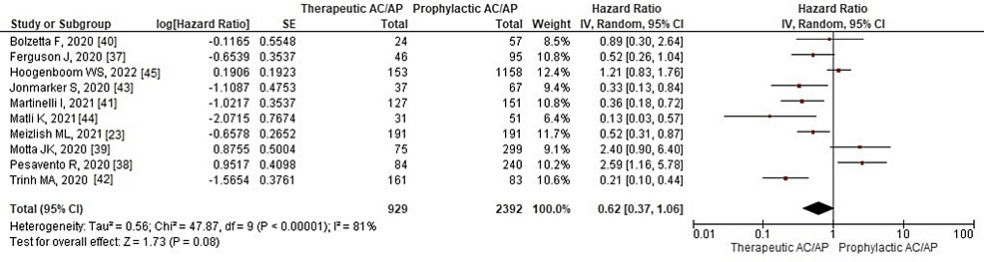
Forest plot of all-cause mortality outcome Red squares and their corresponding lines are the point estimates and 95% CI per each study. Black diamonds represent the pooled effect estimate. IV: inverse variance, SE: standard error, CI: confidence interval, AC: anticoagulant, AP: antiplatelet

All-cause mortality: All 10 studies reported mortality as their outcomes. The usage of the therapeutic dose does not show a significant decrease in mortality than the prophylactic dose (RR: 0.62, 95% CI: 0.37-1.06; p = 0.08; I^2^ = 81%) (Figure 6).

On sensitivity analysis, removing any study did not significantly decrease the heterogeneity value.

Discussion

Our meta-analysis comprising 34 adjusted data studies and a sample size of 287,486 patients found that any dose of AC/AP significantly lowered the mortality risk in COVID-19 patients as compared to patients who received no therapy, with the subgroup analysis demonstrating that the decrease was significant in the AC group compared to the AP group. Subgroup analysis showed that any dose of AC therapy significantly decreased the need for mechanical ventilation in COVID-19-positive patients, while any dose of AP agents yielded no significant results. Patients treated in the any dose of AC/AP group did not have a reduced risk of hospital admissions due to COVID-19. Importantly, our results also reveal that the therapeutic dose of AP/AC was associated with a lower risk of mortality compared to the prophylactic dose, but the results were non-significant.

Our findings align with those of a prior systematic review and meta-analysis conducted by McBane et al., which found that anticoagulation reduced mortality in COVID-19 patients compared to those who did not receive it [7]. However, when comparing mechanical ventilation, our results differ from those of McBane et al.’s study since our analysis shows that anticoagulation significantly reduced the need for mechanical ventilation in COVID-19-positive individuals, compared to their meta-analysis, which reported the contrary. This significant difference can be attributed to the observational nature of the research employed in McBane et al.’s meta-analysis and the small sample size [7]. Previously conducted meta-analyses evaluating the role of antiplatelet therapy for COVID-19 patients show conflicting results, with some studies demonstrating that there is no appreciable decline in mortality following monotherapy with antiplatelet drugs [46-48], while another meta-analysis found that aspirin, an antiplatelet drug, significantly lowered the risk of mortality when used for COVID-19-infected patients (RR: 0.56, 95% CI: 0.38-0.81, p = 0.002) [49].

Thrombosis is a key feature of the SARS-CoV-2 infection and is characterized by an inflammatory response to the virus, endothelium infection, activation, damage, and hypercoagulability [50]. Considering the thrombogenic nature of the virus, current guidelines recommend that all patients admitted with COVID-19 should receive prophylaxis or therapeutic anticoagulation for venous thromboembolism unless they present a bleeding risk [51]. The findings of our meta-analysis also corroborate the efficacy of anticoagulants in the treatment of COVID-19. The infection has also been associated with platelet-derived pro-inflammatory cytokine release, which contributes to hypercoagulability [52]. Hence, antiplatelet agents can also lower the mortality rate in COVID-19 patients [52], although current evidence does not suggest a significant mortality benefit associated with the use of antiplatelet agents [46-48].

Previous data show that patients with COVID-19 admitted to the ICU have a high rate of requiring mechanical ventilation, with a percentage as high as 89.9% in a study. This is concerning as the fatality for patients needing mechanical ventilation was found to be 88% [53] and emphasizes the importance of a treatment agent that would reduce the rate of hospital admission and the need for mechanical ventilation in COVID-19 patients. Therapeutic anticoagulation and antiplatelet therapy or monotherapy with tAC have been associated with better outcomes for patients hospitalized for COVID-19 [44]. While our results show that the AP/AC group did not have a significant advantage over the control group in terms of mechanical ventilation, the subgroup analysis supports that anticoagulants significantly decrease the mechanical ventilation rate in patients admitted to the hospital. Currently, there is a paucity of clinical trials evaluating the association between mechanical ventilation with COVID-19 and anticoagulation/antiplatelet therapy. More clinical trials are required to fully understand this relationship as it could drastically change the course of management of hospitalized patients.

The effectiveness of therapeutic versus prophylactic anticoagulation in the treatment of COVID-19 has also been compared in numerous research. A study shows that tAC does not have superiority over pAC, with the former also being linked with an increased risk of bleeding [5]. Another demonstrates that there is no significant difference in the mortality of tAC and pAC, findings which are consistent with the results of our meta-analysis [54].

To the best of our knowledge, this is the most comprehensive meta-analysis on adjusted data evaluating the relation between AC/AP in COVID-19 and their effect on mortality, hospital admission, and mechanical ventilation. The present analysis has several strengths. Firstly, it has a large sample size of 287,486 patients pooled from 33 studies conducted all across the globe, making the outcomes of our meta-analysis clinically significant. Secondly, there is no publication bias, as indicated by Egger’s test, and the included studies exhibit a low to moderate risk of bias. Implementing adjusted data in our analysis has lowered the risk of confounding variables. The high heterogeneity in our analyses can be explained by the difference in sample sizes among the studies. However, our research also has a few limitations. While we could incorporate three outcomes in Group A (AC/AP versus none), Group B evaluated the efficacy of tAC/AP versus pAC/AP, which only had mortality as its outcome since data on hospital admission and mechanical ventilation was unavailable for this group. Moreover, we were not able to divide the doses quantitatively and accept the primary studies’ definition of therapeutic versus prophylactic doses. A meta-analysis that takes into account the doses in numerical form and analyzes the data would yield more detailed results, but it was beyond the scope of our study.

## Conclusions

Infection with SARS-CoV-2 is associated with a thrombogenic state and hypercoagulability. This can lead to worse outcomes and increased risk of thromboembolic events such as venous thrombosis, and need for mechanical ventilation, and mortality. Many drugs are being employed to improve outcomes in COVID-19 patients. Treatment with AC and AP drugs can significantly decrease the mortality rate in COVID-19-infected patients. In our study, only the AC group significantly reduces the need for mechanical ventilation. Further studies are needed to assess the efficacy of AP in reducing the need for mechanical ventilation. Since the therapeutic dose of AC/AP does not yield major benefits over the prophylactic dose, the prophylactic dose of AP/AC can be used as a first-line treatment for managing thrombotic events, owing to its efficacy in combating the thrombogenesis seen in COVID-19. This could prove to form a basis for further exploration of administering AC and AP drugs in decreasing number of deaths in patients who are suffering from respiratory illnesses.

## Appendices

Table 3 presents a detailed literature search in Google Scholar, PubMed, Medline, Cochrane CENTRAL, and Medrix databases.

**Table 3:** Detailed search strategy

Search engine	Thread
Google Scholar	COVID-19 OR Coronavirus OR SARS-CoV-2 OR severe acute respiratory syndrome coronavirus 2 AND Anticoagulants OR Antiplatelets OR Aspirin OR Warfarin OR Coumadin OR Heparin OR Clopidogrel OR Dabigatran OR Ticagrelor OR Prasugrel OR Acetylsalicylic acid OR Rivaroxaban
PubMed and Medline	(("covid 19"[All Fields] OR "covid 19"[MeSH Terms] OR "covid 19 vaccines"[All Fields] OR "covid 19 vaccines"[MeSH Terms] OR "covid 19 serotherapy"[All Fields] OR "covid 19 serotherapy"[Supplementary Concept] OR "covid 19 nucleic acid testing"[All Fields] OR "covid 19 nucleic acid testing"[MeSH Terms] OR "covid 19 serological testing"[All Fields] OR "covid 19 serological testing"[MeSH Terms] OR "covid 19 testing"[All Fields] OR "covid 19 testing"[MeSH Terms] OR "sars cov 2"[All Fields] OR "sars cov 2"[MeSH Terms] OR "severe acute respiratory syndrome coronavirus 2"[All Fields] OR "ncov"[All Fields] OR "2019 ncov"[All Fields] OR (("coronavirus"[MeSH Terms] OR "coronavirus"[All Fields] OR "cov"[All Fields]) AND 2019/11/01:3000/12/31[Date - Publication]) OR ("coronavirus"[MeSH Terms] OR "coronavirus"[All Fields] OR "coronaviruses"[All Fields]) OR ("sars cov 2"[MeSH Terms] OR "sars cov 2"[All Fields] OR "sars cov 2"[All Fields]) OR ("sars cov 2"[MeSH Terms] OR "sars cov 2"[All Fields] OR "severe acute respiratory syndrome coronavirus 2"[All Fields])) AND ("anticoagulants"[Pharmacological Action] OR "anticoagulants"[MeSH Terms] OR "anticoagulants"[All Fields] OR "anticoagulant"[All Fields] OR "anticoagulate"[All Fields] OR "anticoagulated"[All Fields] OR "anticoagulating"[All Fields] OR "anticoagulation"[All Fields] OR "anticoagulations"[All Fields] OR "anticoagulative"[All Fields])) OR ("antiplatelet"[All Fields] OR "antiplatelets"[All Fields]) OR ("aspirin"[MeSH Terms] OR "aspirin"[All Fields] OR "aspirins"[All Fields] OR "aspirin s"[All Fields] OR "aspirine"[All Fields]) OR ("warfarin"[MeSH Terms] OR "warfarin"[All Fields] OR "warfarin s"[All Fields] OR "warfarinization"[All Fields] OR "warfarinized"[All Fields] OR "warfarins"[All Fields]) OR ("warfarin"[MeSH Terms] OR "warfarin"[All Fields] OR "coumadin"[All Fields] OR "warfarin s"[All Fields] OR "warfarinization"[All Fields] OR "warfarinized"[All Fields] OR "warfarins"[All Fields]) OR ("heparin"[MeSH Terms] OR "heparin"[All Fields] OR "heparine"[All Fields] OR "heparins"[All Fields] OR "heparin s"[All Fields] OR "heparinate"[All Fields] OR "heparinated"[All Fields] OR "heparines"[All Fields] OR "heparinic"[All Fields] OR "heparinisation"[All Fields] OR "heparinised"[All Fields] OR "heparinization"[All Fields] OR "heparinize"[All Fields] OR "heparinized"[All Fields] OR "heparinizing"[All Fields]) OR ("clopidogrel"[MeSH Terms] OR "clopidogrel"[All Fields] OR "clopidogrel s"[All Fields]) OR ("dabigatran"[MeSH Terms] OR "dabigatran"[All Fields] OR "dabigatran s"[All Fields]) OR ("ticagrelor"[MeSH Terms] OR "ticagrelor"[All Fields]) OR ("prasugrel hydrochloride"[MeSH Terms] OR ("prasugrel"[All Fields] AND "hydrochloride"[All Fields]) OR "prasugrel hydrochloride"[All Fields] OR "prasugrel"[All Fields] OR "prasugrel s"[All Fields]) OR ("aspirin"[MeSH Terms] OR "aspirin"[All Fields] OR ("acetylsalicylic"[All Fields] AND "acid"[All Fields]) OR "acetylsalicylic acid"[All Fields]) OR ("rivaroxaban"[MeSH Terms] OR "rivaroxaban"[All Fields])
Cochrane CENTRAL	COVID-19 OR Coronavirus OR SARS-CoV-2 OR severe acute respiratory syndrome coronavirus 2 AND Anticoagulants OR Antiplatelets OR Aspirin OR Warfarin OR Coumadin OR Heparin OR Clopidogrel OR Dabigatran OR Ticagrelor OR Prasugrel OR Acetylsalicylic acid OR Rivaroxaban
Medrix	SARS-CoV-2 OR COVID-19 OR severe acute respiratory syndrome coronavirus 2 OR Coronavirus AND Anticoagulants OR Antiplatelets OR Aspirin OR Warfarin OR Coumadin OR Heparin OR Clopidogrel OR Dabigatran OR Ticagrelor OR Prasugrel OR Acetylsalicylic acid OR Rivaroxaban

## References

[1] (2023). Worldometer: Coronavirus death toll and trends. https://www.worldometers.info/coronavirus/coronavirus-death-toll/.

[2] Long B, Brady WJ, Koyfman A, Gottlieb M (2020). Cardiovascular complications in COVID-19. Am J Emerg Med.

[3] Abou-Ismail MY, Diamond A, Kapoor S, Arafah Y, Nayak L (2020). The hypercoagulable state in COVID-19: incidence, pathophysiology, and management. Thromb Res.

[4] Billett HH, Reyes-Gil M, Szymanski J (2020). Anticoagulation in COVID-19: effect of enoxaparin, heparin, and apixaban on mortality. Thromb Haemost.

[5] Lopes RD, de Barros E Silva PG, Furtado RH (2021). Therapeutic versus prophylactic anticoagulation for patients admitted to hospital with COVID-19 and elevated D-dimer concentration (ACTION): an open-label, multicentre, randomised, controlled trial. Lancet.

[6] Hadid T, Kafri Z, Al-Katib A (2021). Coagulation and anticoagulation in COVID-19. Blood Rev.

[7] McBane RD 2nd, Torres Roldan VD, Niven AS (2020). Anticoagulation in COVID-19: a systematic review, meta-analysis, and rapid guidance from Mayo Clinic. Mayo Clin Proc.

[8] Nicholas K, Yeatts SD, Zhao W, Ciolino J, Borg K, Durkalski V (2015). The impact of covariate adjustment at randomization and analysis for binary outcomes: understanding differences between superiority and noninferiority trials. Stat Med.

[9] Hutton B, Salanti G, Caldwell DM (2015). The PRISMA extension statement for reporting of systematic reviews incorporating network meta-analyses of health care interventions: checklist and explanations. Ann Intern Med.

[10] Higgins JP, Altman DG, Gøtzsche PC (2011). The Cochrane Collaboration's tool for assessing risk of bias in randomised trials. BMJ.

[11] Wells G, Shea B, O’Connell D, Peterson J, Welch V, Losos M, Tugwell P (2000). The Newcastle-Ottawa scale (NOS) for assessing the quality of non-randomized studies in meta-analysis. Appl Eng Agric.

[12] Higgins JP, Thompson SG (2002). Quantifying heterogeneity in a meta-analysis. Stat Med.

[13] Paranjpe I, Fuster V, Lala A (2020). Association of treatment dose anticoagulation with in-hospital survival among hospitalized patients with COVID-19. J Am Coll Cardiol.

[14] Russo V, Di Maio M, Attena E (2020). Clinical impact of pre-admission antithrombotic therapy in hospitalized patients with COVID-19: a multicenter observational study. Pharmacol Res.

[15] Tremblay D, van Gerwen M, Alsen M (2020). Impact of anticoagulation prior to COVID-19 infection: a propensity score-matched cohort study. Blood.

[16] Nadkarni GN, Lala A, Bagiella E (2020). Anticoagulation, bleeding, mortality, and pathology in hospitalized patients with COVID-19. J Am Coll Cardiol.

[17] Shankaranarayanan D, Muthukumar T, Barbar T (2020). Anticoagulation strategies and filter life in COVID-19 patients receiving continuous renal replacement therapy: a single-center experience. Clin J Am Soc Nephrol.

[18] Rivera-Caravaca JM, Núñez-Gil IJ, Vivas D (2021). Clinical profile and prognosis in patients on oral anticoagulation before admission for COVID-19. Eur J Clin Invest.

[19] Rentsch CT, Beckman JA, Tomlinson L (2020). Early initiation of prophylactic anticoagulation for prevention of COVID-19 mortality: a nationwide cohort study of hospitalized patients in the United States. medRxiv.

[20] Denas G, Gennaro N, Ferroni E (2021). Reduction in all-cause mortality in COVID-19 patients on chronic oral anticoagulation: a population-based propensity score matched study. Int J Cardiol.

[21] Flam B, Wintzell V, Ludvigsson JF, Mårtensson J, Pasternak B (2021). Direct oral anticoagulant use and risk of severe COVID-19. J Intern Med.

[22] Meizlish ML, Goshua G, Liu Y (2021). Intermediate-dose anticoagulation, aspirin, and in-hospital mortality in COVID-19: a propensity score-matched analysis. Am J Hematol.

[23] Di Castelnuovo A, Costanzo S, Antinori A (2021). Heparin in COVID-19 patients is associated with reduced in-hospital mortality: the multicenter Italian CORIST study. Thromb Haemost.

[24] Ionescu F, Grasso-Knight G, Castillo E (2020). Therapeutic anticoagulation delays death in COVID-19 patients: cross-sectional analysis of a prospective cohort. TH Open.

[25] Falcone M, Tiseo G, Barbieri G (2020). Role of low-molecular-weight heparin in hospitalized patients with severe acute respiratory syndrome coronavirus 2 pneumonia: a prospective observational study. Open Forum Infect Dis.

[26] Ionescu F, Jaiyesimi I, Petrescu I (2021). Association of anticoagulation dose and survival in hospitalized COVID-19 patients: a retrospective propensity score-weighted analysis. Eur J Haematol.

[27] Chow JH, Khanna AK, Kethireddy S (2021). Aspirin use is associated with decreased mechanical ventilation, intensive care unit admission, and in-hospital mortality in hospitalized patients with coronavirus disease 2019. Anesth Analg.

[28] Al-Samkari H, Gupta S, Leaf RK (2021). Thrombosis, bleeding, and the observational effect of early therapeutic anticoagulation on survival in critically ill patients with COVID-19. Ann Intern Med.

[29] Haji Aghajani M, Moradi O, Amini H, Azhdari Tehrani H, Pourheidar E, Rabiei MM, Sistanizad M (2021). Decreased in-hospital mortality associated with aspirin administration in hospitalized patients due to severe COVID-19. J Med Virol.

[30] Rivera-Caravaca JM, Buckley BJ, Harrison SL, Fazio-Eynullayeva E, Underhill P, Marín F, Lip GY (2021). Direct-acting oral anticoagulants use prior to COVID-19 diagnosis and associations with 30-day clinical outcomes. Thromb Res.

[31] The OpenSAFELY Collaborative, Angel YS Wong, Laurie Tomlinson (2021). Association between oral anticoagulants and COVID-19 related outcomes: two cohort studies. medRxiv.

[32] Vaughn VM, Yost M, Abshire C (2021). Trends in venous thromboembolism anticoagulation in patients hospitalized with COVID-19. JAMA Netw Open.

[33] Spiegelenberg JP, van Gelder MM, Maas ML (2021). Prior use of therapeutic anticoagulation does not protect against COVID-19 related clinical outcomes in hospitalized patients: a propensity score-matched cohort study. Br J Clin Pharmacol.

[34] Lund LC, Hedberg P, Andreasen AH (2022). Prophylactic anticoagulation with low molecular weight heparin in COVID-19: cohort studies in Denmark and Sweden. Clin Microbiol Infect.

[35] Buenen AG, Sinkeldam M, Maas ML, Verdonschot M, Wever PC (2021). Prior use of anticoagulation is associated with a better survival in COVID-19. J Thromb Thrombolysis.

[36] Hara H, Uemura Y, Hayakawa K (2021). Evaluation of the efficacy of anticoagulation therapy in reducing mortality in a nationwide cohort of hospitalized patients with coronavirus disease in Japan. Int J Infect Dis.

[37] Ferguson J, Volk S, Vondracek T, Flanigan J, Chernaik A (2020). Empiric therapeutic anticoagulation and mortality in critically ill patients with respiratory failure from SARS-CoV-2: a retrospective cohort study. J Clin Pharmacol.

[38] Pesavento R, Ceccato D, Pasquetto G (2020). The hazard of (sub)therapeutic doses of anticoagulants in non-critically ill patients with Covid-19: the Padua province experience. J Thromb Haemost.

[39] Motta JK, Ogunnaike RO, Shah R (2020). Clinical outcomes with the use of prophylactic versus therapeutic anticoagulation in coronavirus disease 2019. Crit Care Explor.

[40] Bolzetta F, Maselli M, Formilan M (2021). Prophylactic or therapeutic doses of heparins for COVID-19 infection? A retrospective study. Aging Clin Exp Res.

[41] Martinelli I, Ciavarella A, Abbattista M (2021). Increasing dosages of low-molecular-weight heparin in hospitalized patients with Covid-19. Intern Emerg Med.

[42] Trinh MA, Chang DR, Govindarajulu US (2020). Therapeutic anticoagulation is associated with decreased mortality in mechanically ventilated COVID-19 patients. medRxiv.

[43] Jonmarker S, Hollenberg J, Dahlberg M (2020). Dosing of thromboprophylaxis and mortality in critically ill COVID-19 patients. Crit Care.

[44] Matli K, Chamoun N, Fares A (2021). Combined anticoagulant and antiplatelet therapy is associated with an improved outcome in hospitalised patients with COVID-19: a propensity matched cohort study. Open Heart.

[45] Hoogenboom WS, Lu JQ, Musheyev B (2022). Prophylactic versus therapeutic dose anticoagulation effects on survival among critically ill patients with COVID-19. PLoS One.

[46] Wang Y, Ao G, Nasr B, Qi X (2021). Effect of antiplatelet treatments on patients with COVID-19 infection: a systematic review and meta-analysis. Am J Emerg Med.

[47] Kow CS, Hasan SS (2021). Use of antiplatelet drugs and the risk of mortality in patients with COVID-19: a meta-analysis. J Thromb Thrombolysis.

[48] Patoulias D, Papadopoulos C, Doumas M (2022). Meta-analysis addressing the efficacy and safety of antiplatelet agents in patients with COVID-19. Am J Cardiol.

[49] Wijaya I, Andhika R, Huang I, Purwiga A, Budiman KY (2021). The effects of aspirin on the outcome of COVID-19: a systematic review and meta-analysis. Clin Epidemiol Glob Health.

[50] Bradbury CA, McQuilten Z (2022). Anticoagulation in COVID-19. Lancet.

[51] Hanff TC, Mohareb AM, Giri J, Cohen JB, Chirinos JA (2020). Thrombosis in COVID-19. Am J Hematol.

[52] Santoro F, Nuñez-Gil IJ, Vitale E (2022). Antiplatelet therapy and outcome in COVID-19: the Health Outcome Predictive Evaluation Registry. Heart.

[53] Wunsch H (2020). Mechanical ventilation in COVID-19: interpreting the current epidemiology. Am J Respir Crit Care Med.

[54] Duo H, Li Y, Sun Y (2022). Effect of therapeutic versus prophylactic anticoagulation therapy on clinical outcomes in COVID-19 patients: a systematic review with an updated meta-analysis. Thromb J.

